# Differentiation analyses of adult suspension mononucleated peripheral blood cells of *Mus musculus*

**DOI:** 10.1186/1478-811X-8-29

**Published:** 2010-10-23

**Authors:** Shahrul Hisham Zainal Ariffin, Intan Zarina Zainol Abidin, Muhammad Dain Yazid, Rohaya Megat Abdul Wahab

**Affiliations:** 1 School of Biosciences and Biotechnology, Faculty of Science and Technology, Universiti Kebangsaan Malaysia, 43600 Bangi, Selangor, Malaysia; 2 Department of Orthodontic, Faculty of Dentistry, Universiti Kebangsaan Malaysia, Jalan Raja Muda Abdul Aziz, 50300 Kuala Lumpur, Malaysia

## Abstract

**Background:**

The purpose of this study is to determine whether isolated suspension mouse peripheral mononucleated blood cells have the potential to differentiate into two distinct types of cells, i.e., osteoblasts and osteoclasts.

**Results:**

Differentiation into osteoblast cells was concomitant with the activation of the *Opn *gene, increment of alkaline phosphatase (ALP) activity and the existence of bone nodules, whereas osteoclast cells activated the *Catk *gene, increment of tartrate resistant acid phosphatase (TRAP) activity and showed resorption activities via resorption pits. Morphology analyses showed the morphology of osteoblast and osteoclast cells after von Kossa and May-Grunwald-Giemsa staining respectively.

**Conclusions:**

In conclusion, suspension mononucleated cells have the potentiality to differentiate into mature osteoblasts and osteoclasts, and hence can be categorized as multipotent stem cells.

## Background

Stem cells, or *mother *cells, are cells with the ability to divide for indefinite periods in time and to give rise to specific cells. The primitive stage of stem cells can be divided into three types; i.e., totipotent, pluripotent and multipotent cells [1,2]. Totipotent cells are the most primitive cells, followed by pluripotent cells. The multipotent cell type is the most differentiated type of stem cells [3]. Osteoblasts and osteoclasts are cells that are responsible for bone formation and resorption respectively [4]. During bone formation, osteoblasts deposit the organic and inorganic matrix, whilst osteoclasts remove bone matrix [5]. Osteoblast and osteoclast cells are originated from different lineage, i.e., osteoblasts arise from mesenchymal stem cells and osteoclasts originate from haematopoietic stem cells [6].

The objective of this study is to determine the potentiality of isolated suspension mononucleated cells to differentiate into osteoblast and osteoclast cells. Osteoblasts were originated from different lineage (mesenchymal stem cells) but osteoclasts were originated from haematopoietic stem cells. This indicates that the isolated mononucleated cells capable to differentiate into more than one type of cells. Our results show that suspension mononucleated cells isolated from peripheral mouse blood have the potential to develop into more than one type of mature cell. The results also demonstrate the plasticity of adult stem cells isolated from peripheral blood. These cells are capable of fully differentiating into osteoblast and osteoclast cells. The presences of osteoblast and osteoclast cells were determined using molecular biology, cells activity and morphology analyses.

## Results and Discussion

### Proliferation of Mononucleated Cells

The heterogenic populations were characterised using cell culture selection. After 15 days cultured in selection medium, majority of differentiated and precursor cells died due to short lifespans, e.g., granulocytes (30-40 minutes in the peripheral blood with a total lifespan of 7-13 days that varied under certain pathological conditions) [7], monocytes (5-7 days) [8] and platelets (3-5 days) [9]. As a result, only primitive mononucleated cells (stem cells) survive after 15 days cultured in selection medium.

Besides differentiation potential, self-renewal is the main property of stem cells. The suspension mononucleated cells population doubling times are 3.1 days [10]. Passages were done every doubling time i.e., 3-4 days. Within 15 days, 4-5 passages were used for each isolates which produce a total cell number of approximately 3-5 × 10^6 ^cells/mL for every isolates which start from approximately 1 × 10^5 ^cells/mL. As conclusion, within 15 days the suspension mononucleated cells were shown to proliferate in *in vitro *conditions.

### Activation of Molecular Markers in Differentiated Mononucleated Cells

Previous studies have demonstrated that there are several specific gene markers for osteoblast cells, such as osteopontin, alkaline phosphatase, osteocalcin and collagen type I (Col1) [11,12]. In this study, molecular analysis was performed to determine *in vitro *differentiation of peripheral blood mononucleated cells into mature osteoblast cells after induction by differentiation factors, i.e., ascorbic acid and β-glycerophosphate, for 14 days.

**Figure 1:**
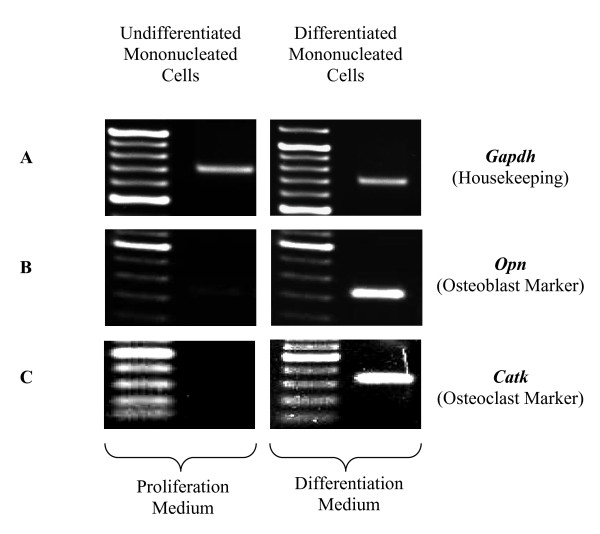
**Expression of specific gene markers**. RT-PCR analysis was performed using total RNA isolated from mononucleated cells cultured in proliferation medium and mononucleated cells cultured in osteoblast and osteoclast differentiation medium. (A) *Gapdh *(717 bp) was used as a positive control. (B) The low expression of *Opn *(234 bp) in undifferentiated mononucleated cells while high expression of *Opn *(234 bp) indicates differentiation into mature osteoblasts. (C) *Catk *(350 bp) expression shows osteoclast differentiation. Equal volumes of the products were separated in agarose gels and stained with ethidium bromide.

The activation of the *Opn *gene shows that mononucleated cells have differentiated into osteoblast cells. RT-PCR analysis showed different levels of *Opn *gene expression in mononucleated cells cultured in proliferation medium compared to osteoblast differentiation medium (Figure 1B). Both media produced the expected products; i.e., the *Opn *gene with size ~234 bp when amplified with RT-PCR. However, the expression of the *Opn *gene for mononucleated cells cultured in osteoblast differentiation medium were much higher as compared to mononucleated cells cultured in proliferation medium (Figure 1B). The increase in *Opn *gene transcription showed that peripheral blood mononucleated cells differentiate into mature osteoblast cells. *Opn *gene can produce low grade expression in variety of cells, such as fibroblast, differentiated osteoblast and osteoclast cells and bone marrow derived cells [13]. Low level expression of *Opn *gene in this study might be due to the existence a few of all these differentiated cells types and also primitive cells that can survive after 15 days of medium selection. However, the increment of expression level after been induced in specialized osteoblast medium was contributed by the primitive cell (stem cells) that have been differentiated into osteoblast cells.

**Figure 2:**
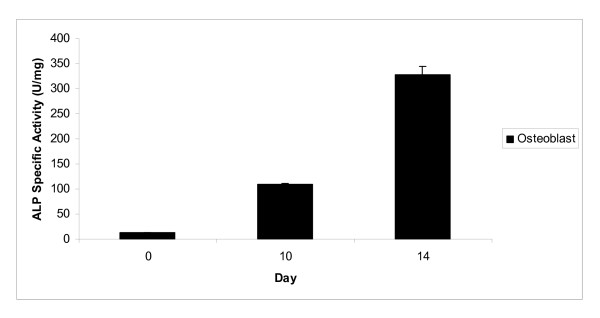
**ALP specific activity for mononucleated cells cultured in osteoblast differentiation medium**. ALP specific activity showed an increment after 14 days mononucleated cells cultured in osteoblast differentiation medium.

RT-PCR analysis was done on day 14 based on the fact that the mononucleated cell line (MC3T3-E1) is fully differentiated into osteoblast cells after 14 days in differentiation medium [14]. Similarly, based on studies in rat calvarial osteoblast-like (ROB) cell, the *Opn *gene was also expressed during 13 to 15 days of differentiation [15]. Furthermore, the study showed that ROB cells differentiate morphologically into mature osteoblast cells after 13 days of differentiation. Therefore, this study demonstrates that mononucleated cells cultured in osteoblast differentiation medium express the *Opn *gene when differentiated into osteoblast cells. In addition, alkaline phosphatase (ALP) assay also showed the increment of ALP activity after 10 and 14 days of mononucleated cells cultured in osteoblast differentiation medium compared to cells cultured in control medium (proliferation medium) (Figure 2). MC3T3-E1 cell line (pre-osteoblast) that cultured in differentiation medium (presence of ascorbic acid and β-glycerophosphate) exhibits high ALP activity after 10 and 14 days in the medium [14,16]. This is an indication that ALP is a marker for the bone formation. The expression of *Opn *gene and increment of ALP activity showed that mononucleated cells are differentiated into osteoblast cells.

In this study, the housekeeping gene *Gapdh *was used as a positive control for mononucleated cells in both types of medium, i.e., proliferation and differentiation medium. Figure 1A shows that RT-PCR amplification produces a *Gapdh *band (~717 bp) from mononucleated cells in both types of media. The activation of the *Gapdh *gene proves that cells perform basic metabolic processes required for cell survival. An inherent assumption in the use of housekeeping genes is that expression of the genes remains constant in the cells or in the tissues under investigation [17].

Differentiated osteoclast cells express specific markers, such as tartrate resistant acid phosphatase and cathepsin K [18,19]. Therefore, the *Catk *gene will only be activated in osteoclast cells. Molecular analysis was performed to determine whether the isolated mononucleated cells had indeed differentiated into mature osteoclast cells. The activation of the *Catk *gene has been observed in mononucleated cells induced *in vitro *for osteoclast differentiation over a period of 10 days. RT-PCR analysis was done on day 10 since mononucleated cells are believed to differentiate into mature osteoclasts at this time point, thus suitable for molecular analysis [20-23].

**Figure 3:**
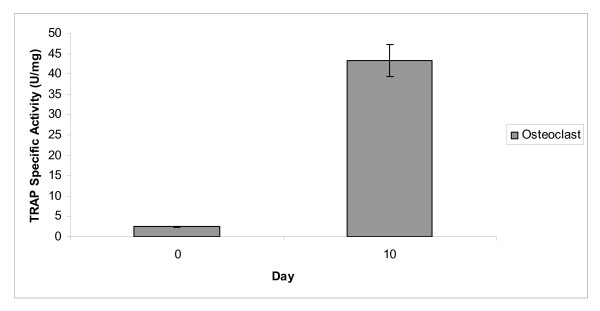
**TRAP specific activity for mononucleated cells cultured in osteoclast differentiation medium**. TRAP specific activity showed an increment after 10 days mononucleated cells cultured in osteoclast differentiation medium.

The experimental results are shown in Figure 1C. The RT-PCR analysis of the transcripts from mononucleated cells cultured in osteoclast differentiation medium showed the activation of the *Catk *gene. The RT-PCR product showed amplification of a band with the expected size for the *Catk *gene, i.e., ~350 bp (Figure 1C). However, no band was found from mononucleated cells cultured in proliferation medium, which served as the negative control experiment (Figure 1C). Therefore, activation of the *Catk *gene does not occur during cells proliferation. This study showed that mononucleated cells cultured in osteoclast differentiation medium for 10 days activate the *Catk *gene, similar to mature osteoclast cells. In addition, the increment of tartrate resistant acid phosphatase (TRAP) activity in mononucleated cells cultured in osteoclast differentiation medium after 10 days compared to cells cultured in control medium (proliferation medium) also indicates that the cells are differentiated into osteoclast cells (Figure 3).

### Osteoblastic and Osteoclastic Cell Activities

**Figure 4:**
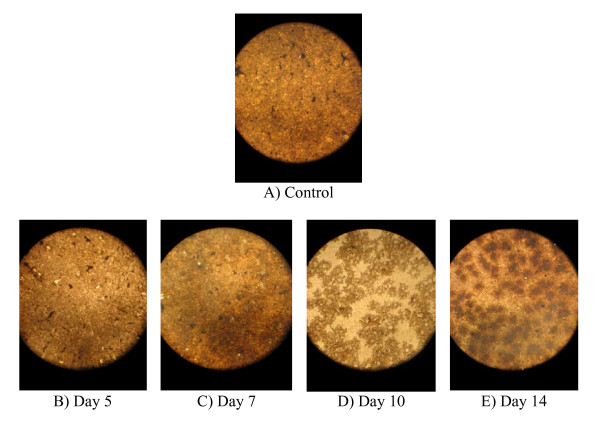
**Osteoblastic activity on osteologic disc in osteoblast differentiation medium**. The discs' background colour became darker each day after von Kossa staining; an indication of the development of bone nodule (B-E) compared to control (A).

Cell activities determination using osteologic discs showed that discs cultured in osteoblast differentiation medium were positive for von Kossa staining, an indication of the existence of calcium nodules (Figure 4). Recent study by Sindrey et al. (1999) showed that generated calcium mineral by osteoblast cells is stained by von Kossa as black or yellow to brown based on the amount of secreted calcium matrices, whereas negative control disc is stained as light brown or gray [24]. The control disc cultured in proliferation medium did not show the formation of calcium nodules on the disc surface (Figure 4A). During differentiation, osteoblasts express the ALP enzyme, which utilizes phosphate from the medium deposits it on the disc surface, which then accumulates to become calcium nodules stained by von Kossa [12,25]. In our study, we compared our discs to the background colour (control disc; cell cultured without osteoblast differentiation factors), where the increment of intensity for the background during cell development has been observed. The background colour and quantity of bone nodules increase starting from day 5 until 14 (Figure 4B-E). Pre-osteoblasts produce high activities after culturing in differentiation medium consisting of ascorbic acid and β-glycerophosphate for 10 and 14 days [14,16].

**Figure 5:**
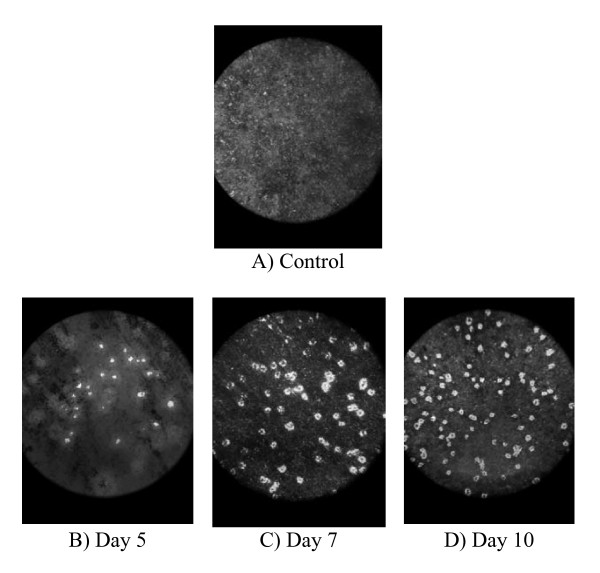
**Osteoclastic activity on osteologic discs in osteoclast differentiation medium**. Resorption pits that appeared clear on the disc surface are increased from day 5 to 10; an indication of mature osteoclast activity (B-D) compared to control (A).

During our osteoclast differentiation assay, the formation of clear resorption pits on the disc surface due to the resorption of the calcium phosphate layer by osteoclast cells was observed (Figure 5). The resorption pits on the disc surface increased from day 5 until 10 (Figure 5B-D). Other studies also showed that osteoclasts are differentiated in culture by day 10 [20-22,26]. Studies by Valverde et al. showed that osteoclastic activity can be determined using bone resorption pits analysis that was performed on calcium phosphate disc (BD BioCoat™ Osteologic™ Disc) [27,28]. Therefore, our study involving similar disc was performed to determine the existence of *in vitro *osteoclastic activity. In addition, there were no resorption pits found on the disc surface for the control disc, indicating the absence of osteoclast activity in cells cultured in proliferation medium (Figure 5A).

### Morphology of Differentiated Osteoblast and Osteoclast Cells

**Figure 6:**
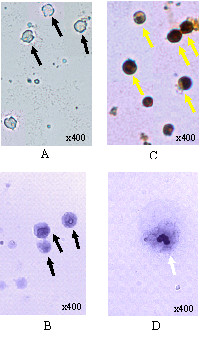
**Morphology of mouse mononucleated cells**. (A and B) Mononucleated cells cultured in proliferation medium stained by von Kossa and May-Grunwald-Giemsa, respectively. (C) Mononucleated cells after 14 days cultured in osteoblast differentiation medium stained by von Kossa. (D) Mononucleated cells after 10 days cultured in osteoclast differentiation medium stained by May-Grunwald-Giemsa. Black arrows show undifferentiated mononucleated cells. Yellow arrows show the black granules in osteoblast cells. White arrow shows multiple nuclei and gigantic osteoclast cells.

The newly isolated mouse peripheral blood mononucleated cells stained by von Kossa (Figure 6A) and May-Grunwald-Giemsa (Figure 6B) were used as controls in this study. These cells were cultured in proliferation medium without any supplementation of differentiation factors. Morphologically, each cell has a large single nucleus that occupies much of the cytoplasmic space (Figures 6A and 6B). The black arrows show the morphology of mononucleated cells without any minerals and calcium deposited when cultured in the proliferation medium.

Figures 6C and 6D show mononucleated cells have been differentiated into osteoblasts and osteoclasts respectively. After von Kossa staining in mononucleated cells cultured in osteoblast differentiation medium for 14 days, mineral nodules were found deposited in these cytoplasmic cells (Figure 6C). According to Alhadlaq and Mao (2003), von Kossa staining can reveal mineral nodules in chondrogenic and osteogenic cells differentiated from mesenchymal stem cells [29]. Other studies also used von Kossa staining to observe the effect of stimulants on osteoblastic cells by quantifying the formation of mineralization nodules in osteoblasts cultures [30]. Therefore, isolated mononucleated cells cultured for 14 days in osteoblast differentiation medium can differentiate into mature osteoblast cells.

Mature osteoclast cells are large multinucleated cells with 6-12 nuclei. Another morphological feature of osteoclasts is the presence of ruffled border and sealing zone [31,32]. May-Grunwald-Giemsa staining showed that these bone-resorbing cells contain many nuclei in its homogenous cytoplasm. May-Grunwald with conjuction of Giemsa is a classic haematology staining procedures that was used to stain peripheral blood and bone marrow specimen. This type of staining can differentiate between nucleus and cytoplasm, which nuclei and cytoplasm as in blue and pink-rose colour, respectively. Osteoclast distinct morphological feature is the presence of multinucleus cells which therefore can be detected using May-Grunwald-Giemsa [33]. After May-Grunwald-Giemsa staining of induced mononucleated cells, the nuclei are purple in colour, while the cytoplasm is lighter than the nucleus (Figure 6D). In this study, mononucleated cells cultured in osteoclast differentiation medium for 10 days showed the morphology of osteoclast cells. The large multinucleated osteoclast cells possessed four nuclei stained purple in colour (Figure 6D). Therefore, mononucleated cells have been differentiated into mature osteoclast cells.

**Figure 7:**
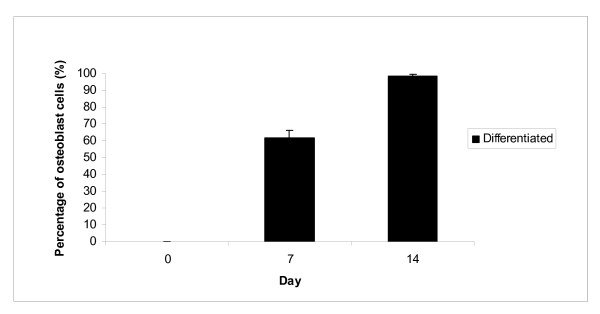
**Morphology analysis of osteoblast differentiation**. Von Kossa staining showed that 62% and 98% of mononucleated cells have been differentiated into osteoblast cells after cultured in osteoblast differentiation medium for 7 and 14 days, respectively.

**Figure 8:**
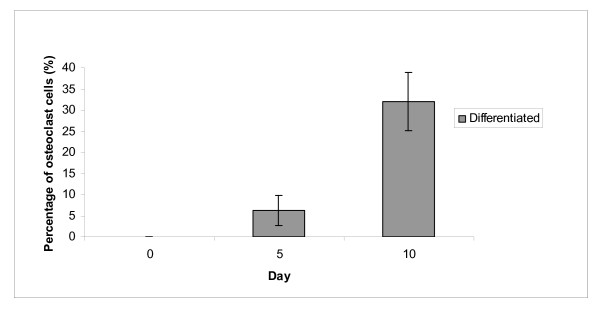
**Morphology analysis of osteoclast differentiation**. May-Grunwald-Giemsa staining showed that 6% and 32% of mononucleated cells have been differentiated into multinucleated osteoclast cells after cultured in osteoblast differentiation medium for 5 and 10 days, respectively.

During differentiation, not all cells are differentiated into osteoblast and osteoclast cells. After 14 days of osteoblast differentiation, there were only about 98% of mononucleated cells are differentiated into osteoblast (Figure 7), while only 32% of mononucleated cells are differentiated into osteoclast cells after 10 days of osteoclast differentiation (Figure 8).

## Conclusions

Mononucleated cells were shown to differentiate into osteoblast and osteoclast cells in their respective differentiation media, as shown by molecular biology, cell activity and morphology analyses. Osteoblast and osteoclast cells originated from different lineages, i.e., osteoblasts from mesenchymal stem cells and osteoclasts from haematopoietic stem cells. The capability of these mononucleated cells to generate different differentiated cells types indicates the plasticity of these adult stem cells. In conclusion, the ability of suspension mononucleated cells isolated from adult mouse peripheral blood to differentiate into both cells lineages demonstrates that these types of cells can be categorised as multipotent stem cells.

## Methods

The animal experimental research has followed the animal guidelines provided by Faculty of Science and Technology, Universiti Kebangsaan Malaysia, which is similar to international animal guidelines.

### Isolation and Proliferation of Mononucleated Cells

Mononucleated cells were isolated from mice by density gradient centrifugation using Ficoll-Paque™ Plus. The isolated cells were cultured in proliferation medium containing Alpha Minimal Essential Medium (AMEM) with 10% heat-inactivated Newborn Calf Serum (NBCS) and 2% penicillin/streptomycin. The cultures were incubated at 37°C in a fully humidified atmosphere containing 5% CO_2_. Each experiment was isolated from one mouse. Therefore for three independent experiments generated from our data were isolated from three different mice.

### Differentiation of Mononucleated Cells

Suspension mononucleated cells were seeded at 1.0 × 10^5 ^cells/mL in 24-well plates for osteoblast and osteoclast differentiation assays. Cells cultured in complete medium were then supplemented with 50 μg/mL ascorbic acid and 10 mM β-glycerophosphate to induce differentiation into osteoblasts, while 50 ng/mL RANKL and 25 ng/mL M-CSF were added to induce osteoclast differentiation. The cultures were maintained at 37°C in a fully humidified atmosphere containing 5% CO_2_. For control, the same cells were cultured with complete medium without supplementation of growth factors. Mononucleated cells viability in both differentiation and control mediums were assessed using trypan blue.

### Molecular Analyses of Differentiated Mononucleated Cells

Peripheral blood mononucleated cells have been isolated from three different mice and RT-PCR was done for each of them. Total RNA was isolated from approximately 1 × 10^5 ^cells that produce approximately 1.6 mg/mL of RNA using TRI-Reagent (Sigma, USA) according to the manufacturer's instructions. Absorbance was measured at 260 and 280 nm to give ratio of A260:A280 was 1.8 to 2.0 in range to determine the samples purity. Total RNA was isolated from cells in differentiation medium and then reverse-transcribed using AMV reverse transcriptase and T*fl *DNA polymerase according to the Access RT-PCR System protocol (Promega Corporation, USA). The primers sequences used were: 1) *Gapdh*, forward:

^5'^CACTCCAATCGTCCCTACA^3' ^and reverse: ^5'^AAGGTGGAAGAGTGGGAG^3'^, 2) *Opn*, forward: ^5'^CACTCCAATCGTCCCTACA^3' ^and reverse: ^5'^GCTGCCCTTTCCGTTGTT^3'^, and 3) *Catk*, forward: ^5'^GGCAGGGTCCCAGACTCCAT^3' ^and reverse: ^5'^GTGTTGGTGGTGGGCTAC^3'^.

First strand cDNA synthesis was done at 45°C for 45 minutes. The pre-denaturation was done at 95°C for 2 minutes. The temperature cycle used to produce the second cDNA strand and RT-PCR amplification for *Gapdh *(denaturation: 94°C, 30 s; annealing: 62°C, 60 s; elongation: 68°C, 60 s), *Opn *(denaturation: 68°C, 60 s; annealing: 62°C, 60 s; elongation: 68°C, 60 s) and *Catk *(denaturation: 95°C, 30 s; annealing: 63°C, 60 s; elongation: 68°C, 60 s) genes. After 40 cycles, elongation of the final strand was done at 68°C for 7 minutes. The RT-PCR products were then analysed via electrophoresis of 1% agarose gels at 85 V using a 100-bp DNA marker (Vivantis). Each RT-PCR product was then subjected for sequencing and analysed.

### Osteoblastic and Osteoclastic Cell Analyses

Cell activities for mononucleated cells cultured in osteoblast and osteoclast differentiation medium were determined using an osteologic disc. An osteologic disc is a disc coated with a micro layer of calcium phosphate on its surface. The cultured discs were stained using von Kossa staining on specific days, i.e., days 5, 7, 10 and 14 for osteoblast differentiation medium and days 5, 7 and 10 for osteoclast differentiation medium. The cell activities on the discs were observed using an inverted microscope (Olympus, Model: CKX75). Images were collected using a digital camera.

### Morphology Analyses of Differentiated Osteoblast and Osteoclast Cells

Cytospin was used to analyze osteoblast and osteoclast morphology in this study. Approximately, 5-10 × 10^5 ^cells/mL mononucleated cells were centrifuged at 78 × g for 5 minutes. The pellet was smeared onto a glass slide and left to air-dry for about 1 to 2 hours. The cells were then stained with von Kossa and May-Grunwald-Giemsa stains to identify osteoblast and osteoclast cells, respectively. The slides were observed using an inverted microscope (Olympus, Model: CKX75). The percentages of differentiated cells were calculated based on a total of 200 cells counted randomly for approximately 5 different fields under microscope for each experiment.

## List of abbreviations

(ALP): Alkaline Phosphatase; (AMEM): Alpha Minimal Essential Medium; (*Catk*): Cathepsin K; (Col1): Collagen type I; (*Gapdh*): Glyceraldehyde-3-phosphate dehydrogenase; (M-CSF): Macrophage Colony-Stimulating Factor; (NBCS): Newborn Calf Serum; (*Opn*): Osteopontin; (PBS): Phosphate Buffer Saline; (RANKL): Receptor Activator NF-κB Ligand; (RT-PCR): Reverse Transcriptase-Polymerase Chain Reactions; (TRAP): Tartrate Resistant Acid Phosphatase.

## Competing interests

The authors declare that they have no competing interests.

## Authors' contributions

SHZA and RMAW developed the concept, designed and supervised the experiments. IZZA performed and developed experimental study and doing the analysis as part of PhD Thesis by research. MDY carried out part of the molecular and morphology analyses. All experiments and analysis were performed by IZZA and MDY, and approved by SHZA and RMAW. All authors read and approved the final manuscript before publication.
